# Transferrin-Conjugated Polymeric Nanoparticle for Receptor-Mediated Delivery of Doxorubicin in Doxorubicin-Resistant Breast Cancer Cells

**DOI:** 10.3390/pharmaceutics11020063

**Published:** 2019-02-01

**Authors:** Zar Chi Soe, Jun Bum Kwon, Raj Kumar Thapa, Wenquan Ou, Hanh Thuy Nguyen, Milan Gautam, Kyung Taek Oh, Han-Gon Choi, Sae Kwang Ku, Chul Soon Yong, Jong Oh Kim

**Affiliations:** 1 College of Pharmacy, Yeungnam University, 214-1, Dae-dong, Gyeongsan 712-749, Korea; zarchisoeygn96@gmail.com (Z.C.S.); kgb0703@hanmail.net (J.B.K.); thapa.rajkumar7@gmail.com (R.K.T.); owqcn@foxmail.com (W.O.); nguyenhanhthuy.87@gmail.com (H.T.N.); gtmmilan2@gmail.com (M.G.); 2 Department of Pharmaceutics, University of Pharmacy (Yangon), Waybargi Road, North Okkalapa township, Yangon 11031, Myanmar; 3 College of Pharmacy, Chung-Ang University, 221 Heuksuk-dong Dongjak-gu, Seoul 156-756, Korea; kyungoh@cau.ac.kr; 4 College of Pharmacy, Institute of Pharmaceutical Science and Technology, Hanyang University, 55 Hanyangdaehak-ro, Sangnok-gu, Ansan 426-791, Korea; hangon@hanyang.ac.kr; 5 College of Korean Medicine, Daegu Haany University, Gyeongsan 712-715, Korea

**Keywords:** doxorubicin, doxorubicin-resistant cancer, polymeric nanoparticles, transferrin

## Abstract

In this study, a transferrin (T_f_)-conjugated polymeric nanoparticle was developed for the targeted delivery of the chemotherapeutic agent doxorubicin (Dox) in order to overcome multi-drug resistance in cancer treatment. Our objective was to improve Dox delivery for producing significant antitumor efficacy in Dox-resistant (R) breast cancer cell lines with minimum toxicity to healthy cells. The results of our experiments revealed that Dox was successfully loaded inside a transferrin (T_f_)-conjugated polymeric nanoparticle composed of poloxamer 407 (F127) and 123 (P123) (Dox/F127*&*P123-T_f_), which produced nanosized particles (~90 nm) with a low polydispersity index (~0.23). The accelerated and controlled release profiles of Dox from the nanoparticles were characterized in acidic and physiological pH and Dox/F127*&*P123-T_f_ enhanced Dox cytotoxicity in OVCAR-3, MDA-MB-231, and MDA-MB-231(R) cell lines through induction of cellular apoptosis. Moreover, Dox/F127*&*P123-T_f_ inhibited cell migration and altered the cell cycle patterns of different cancer cells. In vivo study in MDA-MB-231(R) tumor-bearing mice demonstrated enhanced delivery of nanoparticles to the tumor site when coated in a targeting moiety. Therefore, Dox/F127*&*P123-T_f_ has been tailored, using the principles of nanotherapeutics, to overcome drug-resistant chemotherapy.

## 1. Introduction

Nanotechnology contributes greatly to the design and development of chemotherapeutic drug formulations to overcome various shortcomings of traditional chemotherapy by improving therapeutic efficacy [1]. Nowadays, cancer treatment strategies involving the development of surface-modified, disease-targeted nanocarriers are being pursued to overcome limitations such as toxicity and multi-drug resistance (MDR) in various cancers [2]. Site-specific and efficient delivery of chemotherapeutic drug is important in MDR and chemotherapeutic drugs can be successfully delivered by passively and actively targeted nanoparticles. Passive targeting strategies increase the intracellular accumulation of chemotherapeutic drugs in a solid tumor environment by enhancing permeability and retention effect (EPR) effect [3]. Actively targeted nanocarriers have beneficial effects in chemotherapeutic drug delivery system such as enhancing drug retention in tumor cells by increasing cellular binding and drug accumulation, and improving cellular uptake by receptor-mediated endocytosis [4].

Polymeric nanomedicines are designed by using amphiphilic organic molecules such as polypeptides, lipids, and block copolymers to encapsulate chemotherapeutic agents, like paclitaxel, cisplatin, and doxorubicin [5]. Polymeric silica nanoparticles (PSNPs) have advantages over other nanoparticles, such as biocompatibility, mechanical robustness, versatility, and the ability to construct nanostructures with large inner cavities and low densities. Moreover, the smaller particle size of the silica shell of a PSNPs causes diffusion of drug molecules from its cavity with perfect colloidal stability [6], and its onion-like structure favors a higher loading capacity and release rate of cargo drugs [7]. PSNPs are extensively studied and represented as drug carriers in polymer-based drug delivery systems because they enhance therapeutic efficacy by increasing endocytosis-mediated drug uptake [8]. Poloxamers can sensitize MDR cancer cells to various chemotherapeutic agents by assimilation into biological membranes and subsequent interference with intracellular functions, mitochondrial respiration, and adenosine triphosphate (ATP) synthesis, which is essential for the action of ATP-binding cassette (ABC) efflux proteins [9]. Moreover, several cancer cells encounter MDR by various mechanisms including overexpression of P-glycoprotein (P-gp) and (ABC) transport proteins [10,11] and the therapeutic dosage regimen can be disturbed, leading to severe untoward effects [12].

The surface of polymeric nanoparticle can be modified by fixing specific homing ligands such as monoclonal antibodies, peptides, nucleic acids, aptamers, and small molecules including epidermal growth factor, folic acid, CD44 or CD22, and transferrin (T_f_) to optimize the active targeting delivery of the chemotherapeutics drugs [13,14,15,16]. The tumor-specific ligands of polymeric nanoparticles trigger receptor-mediated endocytosis and internalization of nanoparticles into cancer cells by interacting with receptors on the surface of cancer cells [14]. T_f_ is a blood plasma glycoprotein known to be an iron transporter and can be used as a targeting site for cancer-specific drug delivery to enhance therapeutic efficacy against various cancer cells that overexpress T_f_ receptors [17]. The half-life, tissue distribution, and drug release in the plasma can be controlled by T_f_-conjugated PSNPs, creating an excellent tool for favoring the accumulation of non-selective chemotherapeutic drugs at targeted areas while reducing the exposure of normal healthy cells to these drugs. In this way, overall therapeutic efficacy has been improved by increasing intracellular concentration and, consequently, anticancer activity [18,19,20].

Doxorubicin (Dox), an anthracycline, is considered one of the most powerful chemotherapeutic agents and is commonly used in multiple cancers, including ovarian and breast cancer. Dox can directly kill tumor cells via DNA damage [21] and induce apoptosis in cancer cells by activation of reactive oxygen species and p53 proteins [22,23]. However, the development of drug resistance in cancer cells remains a major hurdle in effective Dox therapy [24]. The ATP-dependent ABC transporters can support drug efflux and bring substrates across biological membranes against concentration gradients. Three well-known ABC transporters, ABCG2/breast cancer resistance protein (BCRP), ABCB1/p-glycoprotein (P-gp), and ABCC1/multidrug resistance-associated protein 1 (MRP1), are responsible for the development of Dox resistance [25,26]. 

**Figure 1:**
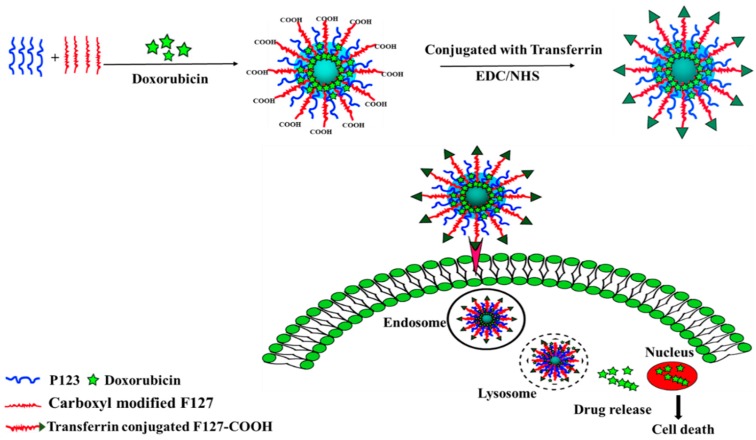
Schematic illustration of the preparation of transferrin-conjugated polymeric nanoparticle for receptor-mediated delivery of Dox in Dox-resistant breast cancer cells.

The aim of this study was to overcome Dox resistance in chemotherapy by using T_f_-targeted PSNPs. To this end, as shown in Figure 1, Dox-loaded poloxamer silica nanoparticles conjugated with T_f_ (Dox/F127&P123-T_f_) were prepared and characterized in vitro. In addition, the in vivo biodistribution of Dox/F127&P123-T_f_ was determined in xenograft mouse models bearing the Dox-resistant breast cancer cell line, MDA-MB-231(R).

## 2. Materials and Methods 

### 2.1. Materials

Dox was obtained from Dong-A Pharmaceutical Company (Yongin, Korea). Poloxamer 407 (Pluronic^®^ F-127, F127), poloxamer P123 (Pluronic^®^ P-123, P123), holo-transferrin (T_f_), 3-(4,5-dimethylthiazol-2-yl)-2,5-diphenyltetrazolium bromide (MTT), 1-ethyl-3-(3-dimethylaminopropyl) carbodiimide hydrochloride (EDC), *N*-hydroxysuccinimide (NHS), and dimethylsulfoxide (DMSO) were obtained from Sigma-Aldrich Co. (St. Louis, MO, USA). Tetramethoxysilane (TMOS) and pyrene were purchased from Aladdin Industrial Corporation (Shanghai, China). The MDA-MB-231 cell line was obtained from the Korean Cell Line Bank (Seoul, Korea). DSPE-PEG_2000_ was purchased from Avanti Polar Lipid (Alabaster, AL, USA). Coumarin-6 and LysoTracker Red were purchased from Thermo Fisher Scientific Inc. (Waltham, MA, USA). Primary antibodies anti-BAX, anti-Bcl-2, anti-caspase-3 and -8, anti-p27, and anti-p53 were purchased from Cell Signaling Technology (Danvers, MA, USA). All other chemicals were of reagent grade and used without further purification.

### 2.2. Development of Dox-Resistant MDA-MB-231 Cells

The modified resistant strain of MDA-MB-231 was developed using the method previously reported by Biki et al. [27]. Briefly, MDA-MB-231 cells were cultured in medium that contained Dox (0.02 μg/mL) and repeatedly cultured with increasing Dox concentrations 1.5 to 2-fold of previous concentration until it reached IC_100_ concentration, 0.2 μg/mL. In each step, dead cells were removed and new Dox concentration was added in fresh media. The resultant MDA-MB-231(R) cells were analyzed to determine characteristics such as efficacy of Dox in this cell line, levels of ABCB1/p-glycoprotein (P-gp) and the apoptosis-stimulating protein p53, and cellular uptake of Dox, and these were compared with those of the original MDA-MB-231 cells (Figure S1). The proliferated cells were deemed to be Dox-resistant and were termed MDA-MB-231(R) cells. These cells were used for further studies involving resistant cancer cells.

### 2.3. Synthesis of Carboxylated Poloxamer 407

Poloxamer 407 (F127) was carboxylated using a modified method [7]. Briefly, F127 (3.5 g) was dissolved in a mixture of anhydrous toluene (15 mL) and pyridine (1 mL). This solution was stirred under an atmosphere of nitrogen and heated to 70 °C. Succinic anhydride (75 mg) was then added and the mixture was refluxed at 90 °C for 3 h. Finally, the mixture was distillated, and the resulting residue was washed with cold ether, then dried at 40 °C for 24 h under a vacuum. The synthesized product was analyzed by Fourier-transform infrared (FTIR) and proton nuclear magnetic resonance spectrometry (^1^H NMR) (Figure S2) to identify the characteristic peaks of F127-COOH. ^1^H NMR spectra were generated by analyzing samples dissolved in d_6_-DMSO using an NMR spectrometer (Varian Inc., Palo Alto, CA, USA).

### 2.4. Preparation of Dox/F127&P123-T_f_

A modified thin-film hydration method with TMOS was used to prepare Dox/F127&P123 and Dox/F127&P123-T_f_ [28,29]. Briefly, a mixture of P123 (150 mg) and carboxylated F127 (100 mg) was dissolved in chloroform and sonicated for 15 min at 25 °C. Dox was added into the F127&P123 solution, which was then sonicated for 20 min. The organic solvent was removed by rotary evaporation and the resultant thin film was hydrated with water and sonicated. A solution of 35 μL TMOS in 0.5 mL tetrahydrofuran (THF) was added dropwise to the above solution while stirring at 1000 rpm, and the solution was further stirred for three days at room temperature to evaporate the THF and hydrolyze the TMOS to form the silica shell. Finally, to get rid of the unbound drug aggregate, the preparation was filtered through a 0.22 μm filtering membrane and the product was kept at 4 °C for subsequent conjugation with T_f_. The amine group of T_f_ was conjugated to the Dox/F127&P123 nanoparticle using an EDC/NHS-based carbodiimide method. Briefly, equivalent amounts of EDC and NHS (0.25 M) were added to Dox/F127&P123 nanoparticle and stirred for 15 min at room temperature. To this solution, 130 μg of T_f_ in PBS (pH 7.4) was added and the mixture was incubated overnight. The unbound protein was separated from the nanoparticle by ultracentrifugation at 93,000g (RCF) for 30 min. A BCA protein assay was used to estimate the coupling efficiency [30].

### 2.5. Morphological Analysis and Characterization of Dox/F127&P123-T_f_

The Z-average hydrodynamic particle size, polydispersity index (PDI), and zeta-potential of nanoparticles were determined using a dynamic light scattering (DLS) method (Nano ZS 90, Zeta Sizer; Malvern Instruments, Malvern, UK) with Nano DTS software, version 6.34 (Malvern Instruments) [31]. Mean values were calculated from three individual measurements.

The morphology of formulated nanoparticles was characterized using a transmission electron microscope (TEM) (H7600; Hitachi, Tokyo, Japan). Briefly, the prepared samples were stained with phosphotungstic acid solution and loaded on carbon-coated copper grids and dried at room temperature. Images were obtained using transmission electron microscopy at 120 kV [32].

To confirm the exact morphology of the nanoparticles, atomic force microscopy (AFM) images were taken using a Nanoscope IIIa scanning probe microscope (Digital Instruments, Murray Hill, NJ, USA). The X-ray diffraction (XRD) patterns of freeze-dried nanocapsules were obtained to identify their crystalline properties using a vertical goniometer and X-ray diffractometer (X’pert PRO MPD diffractometer, Almelo, The Netherlands) [33] at 25 °C with a diffraction angle (2θ) range of 10°–60°, scanning rate of 5°/min, voltage of 40 kV, and current of 30 mA. Furthermore, the FTIR spectrum of Dox/F127&P123-T_f_ was compared with the spectra of Dox, F127&P123 nanoparticles and Dox/F127&P123-T_f_ using a ‘Thermo Scientific Nicolet Nexus 670 FTIR spectrophotometer [34].

### 2.6. Entrapment Efficiency and Loading Capacity of Dox in Dox/F127&P123-T_f_

To calculate the entrapment efficiency (EE) and loading capacity (LC) of Dox in Dox/F127&P123-T_f_, an Amicon centrifugal filter device (molecular weight cut-off of 10,000 Da, Millipore) was used [35]. The formulation was centrifuged at 23,000g (RCF) for 10 min to separate unbound drug, and the resultant filtrate was analyzed using an HPLC system (Hitachi, Japan) comprised of an L-2130 pump, L-2200 autosampler, L-2420 UV-Vis detector, and L-2350 column oven and equipped with EZChrom Elite software (318a, Tokyo, Japan) [36]. For isocratic elution with a mobile phase comprising of methanol: water: acetic acid (50:49:1 *v*/*v*, pH 3.0), an Inertsil C_18_ column (150 mm × 4.6 mm, 5 μm particle size; Cosmosil, Nacalai Tesque Inc., San Diego, CA, USA) was used at a flow rate of 1.0 mL/min and column temperature of 25 °C. A 20-μL sample was injected for each analysis and the absorbance was measured at a wavelength of 480 nm. The percentages of EE and LC were calculated according to the following equation:

(1) $$EE\;\left(\%\right)=\frac{W_{D}}{W_{T}}\times 100,$$

where *W*_D_ is the weight of Dox encapsulated in Dox/F127&P123-T_f_ and *W*_T_ is the total weight of Dox in the formulation; and

(2) $$LC\left(\%\right)=\frac{W_{\mathrm{TD}}-W_{\mathrm{UD}}}{W_{\mathrm{TN}}}\times 100,$$

where *W*_TD_, *W*_UD_, and *W*_TN_ are the weights of total Dox, unbound Dox, and total F127&P123 nanoparticle, respectively.

### 2.7. In Vitro Drug Release Study

To assess the in vitro release of Dox from Dox/F127&P123 and Dox/F127&P123-T_f_, a predetermined volume of formulation was added to a Spectra/Por 3500 Da-MWCO membrane tube and immersed in 30 mL of either acetate-buffered saline (ABS, 0.01 M, pH 5.0) or phosphate-buffered saline (PBS, 0.01 M, pH 6.5 and pH 7.4), according to the most versatile and popular method, the dialysis method. The experiment was performed at 100 rpm and 37 °C. Required samples were withdrawn at specified times, and buffer was replaced with fresh media. The extent of drug release was determined using HPLC as reported previously.

### 2.8. In Vitro Cellular Uptake of Dox/F127&P123-T_f_

To determine quantitative cellular uptake of F127&P123 PSNPs and Dox/F127&P123-T_f_ in cancer cell lines, fluorescence-activated cell sorting (FACS) analysis was performed. OVCAR-3, MDA-MB-231, and MDA-MB-231(R) cells (2 × 10^5^ cells per well) were seeded into 6-well plates and incubated for 24 h [37]. Untreated cells were used as a control. To confirm the uptake efficiency of Dox/F127&P123-T_f_ in Dox-resistant MDA-MB-231 cells, all cell lines were treated with free Dox (100 µg/mL) and Dox/F127&P123-T_f_. After incubation period (1 h), the cells were washed with cold PBS and uptake of Dox and Dox/F127&P123-T_f_ in individual cells was determined using FACS Verse (BD Biosciences, San Jose, CA, USA). The uptake of the nanoparticle in all cell lines was also determined using the same procedure. The role of T_f_ in uptake was determined by pretreating with T_f_ in one group of cells from each cell line before treatment with each test formulation [38].

### 2.9. Cell Migration Efficacy of Dox/F127&P123-T_f_

To examine the anti-metastatic properties of each formulation, a scratch wound assay was performed in all three cell lines. The straight cell-free ‘scratch’ was created on monolayer of cells using a 20 µL pipette tip and washed twice with 1X PBS after all cells had been seeded (1 × 10^5^ /well) in a 12-well plate. Free Dox (100 µg/mL), Dox/F127&P123, and Dox/F127&P123-T_f_ were added to appropriate wells and incubated. After 24 h of treatment, the images of wound edges were recorded using a fluorescence microscope (Nikon Eclipse Ti, Nikon Instruments Inc.; Melville, NY, USA) and contrasted with conditions before treatment [39].

### 2.10. In Vitro Cell Cytotoxicity

To screen for the cytotoxic effects of formulations in different cell lines, an MTT assay was carried out [31] on cells treated with free Dox, Dox/F127&P123, and Dox/F127&P123-T_f_. After cells had been seeded into 96-well plates at a density of 1 × 10^4^ cells per well, all cells were then incubated for 24 h with the appropriate treatment. Finally, 100 µL of tetrazolium dye MTT solution containing 1.25 mg/mL of MTT was added to reacted cells and the absorbance measured at 570 nm using an automated microplate reader. Untreated cells were used as a control. The percentage of viable cells in each treatment was calculated with the following formula:

(3) $$Cell\;viability\;\left(\%\right)=\frac{A570\left(sample\right)-A570\left(blank\right)}{A570\;\left(control\right)-A570\left(blank\right)}\times 100,$$

where OD is the optical density.

### 2.11. Live and Dead Cell Assay of Dox/F127&P123-T_f_

The potential cytotoxic effects of Dox/F127&P123-T_f_ on folate receptor-expressing cells was analyzed using a live/dead assay [40,41]. Individual cells at a density of 1 × 10^5^ cells per well in a 12-well plate were incubated for 24 h. After 24 h, cells were incubated with Dox, Dox/F127&P123, or Dox/F127&P123-T_f_ for a further 24 h. All treated cells were washed with PBS then stained with green (acridine orange) and red fluorescence (propidium iodide) and imaged using fluorescence microscopy (Nikon Eclipse Ti).

### 2.12. Cell Cycle Arrest of Dox/F127&P123-T_f_

To estimate the relative percentages of treated cells in the G_1_, S, G_2_, and M phases of the mammalian cell cycle in, cell cycle analysis was carried out using the Cell-Clock^TM^ Assay kit (Biocolor Ltd., Carrickfergus, UK). All seeded cells (1 × 10^5^ cells per well) in a 12-well plate were treated with free Dox, Dox/F127&P123, or Dox/F127&P123-T_f_ and incubated. After 24 h, incubated cells were stained with redox dye (Cell-Clock Dye Reagent) for 1 h at 37 °C. Images were taken by fluorescence microscope (Nikon Eclipse Ti) and the percentage of cells arrested in each phase was estimated from digitized photomicrographs using ImageJ software 1.46jar [42].

### 2.13. Western Blot Analysis

To detect specific apoptosis-related protein levels in all treated cell lines, Western blot analysis was carried out using previously described methods [43]. Briefly, proteins were extracted after treatment with free Dox, Dox/F127&P123, and Dox/F127&P123-T_f_ and protein concentrations were quantified using the BSA Protein Assay Kit (Thermo Scientific, Waltham, IL, USA). All proteins were separated on 10% Bis-Tris polyacrylamide gel (at 210 mA for 120 min) and transferred to polyvinyl fluoride membrane. The membranes were blocked for 1 h with 5% nonfat milk powder suspension in TBST and incubated overnight with purified primary antibodies: anti-Bax, anti-Bcl-2, anti-p53, anti-p27, anti-caspase-3, and anti-caspase-8. The samples were incubated with suitable secondary antibodies for 1 h, then protein bands were examined with a luminol solution (Thermo Fisher Scientific) and photographed using enhanced chemiluminescence.

### 2.14. In Vivo Imaging and Biodistribution Analysis

To track nanoparticle distribution in the bodies of experimental animals, F127&P123 PSNPs with or without the targeting ligand were injected into the tail veins of BALB/c nude mice bearing MDA-MB-231(R) xenografts with cyanine 5.5 as the fluorescent dye. Fluorescence signals were observed at 6, 12, and 24 h using a FOBI fluorescence imaging system (Neoscience Co. Ltd., Suwon, Korea) [44]. After 24 h, organs (tumor, heart, lung, spleen, kidney, and liver) were collected from sacrificed mice and the fluorescence intensity quantified. The experiment was approved by the Institutional Animal Ethical Committee, Yeungnam University, South Korea (approval number: 2015-008).

### 2.15. Statistical Analysis

To evaluate the statistical differences between the groups, one-way analysis of variance (ANOVA) and Student’s *t*-test (for pairs of groups) were used. *P* < 0.05 was considered statistically significant. All the obtained experimental results are expressed as mean ± standard deviation (SD).

## 3. Results and Discussion

### 3.1. Physicochemical Characterization of Dox/F127&P123-T_f_ NPs

**Figure 2:**
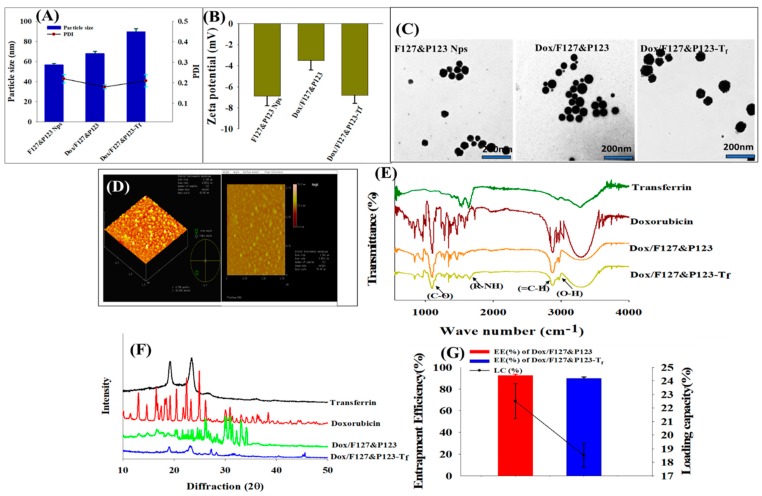
(**A**) Particle sizes and polydispersity indexes (PDIs), (**B**) zeta potentials, and (**C**) TEM images of F127&P123 NPs, Dox/F127&P123, and Dox/F127&P123-T_f_; (**D**) AFM images of Dox/F127&P123-T_f_; (**E**) FTIR spectra, and (**F**) X-ray diffraction patterns of Dox, T_f_, Dox/F127&P123, and Dox/F127&P123-T_f_; (**G**) entrapment efficiency (EE) and loading capacity (LC) of Dox in Dox/F127&P123, and Dox/F127&P123-T_f_.

The average particle sizes of Dox/F127&P123 and Dox/F127&P123-T_f_ were 72.5 ± 1.5 nm and 90.8 ± 2.1 nm, respectively with polydispersity indexes (0.170 ± 0.005 and 0.190 ± 0.004, respectively) suggesting uniform particle size distribution (Figure 2A). The zeta potential of the T_f_-conjugated nanoparticles (‒16.5 ± 0.9 mV) was slightly more negative than that of Dox/F127&P123 NPs (−9.8 ± 1.2 mV) (Figure 2B). The net charge of the nanoparticles may be affected by modifications to T_f_ or the loss of the amine group potentially causing the negatively charged transferrin to conjugate to the surface of the polymeric NPs [45]. TEM imagery revealed that the NPs had spherical appearance and good size distribution. A thin outer layer on the Dox-loaded NPs that were coated with modified T_f_ was observed in the TEM images (Figure 2C). Moreover, AFM imaging (Figure 2D) showed typical morphological characters such as island-like structures on the surface of the NPs, corresponding to the thickness and diameter of Dox/F127&P123-T_f_ NPs [46].

Functional groups and covalent bonding in Dox/F127&P123-T_f_ were characterized by the prominent peaks in FTIR spectra of Dox/F127&P123-T_f_, as seen in Figure 2E. The (‒OH) stretching vibration at 3098 cm^−1^ corresponds to the functional groups responsible for negative charges on NPs. Moreover, bands for the (C‒O) and (=C‒H) groups arising from Dox appeared at 1119 cm^−1^ and 2891 cm^−1^, respectively. Finally, an amide band was observed at 1665 cm^−1^, corresponding to the amide group of T_f_, proving that the surface of Dox/F127&P123-T_f_ was successfully coated in T_f_ [47].

There was clear evidence that Dox/F127&P123-T_f_ was less crystalline than Dox/F127&P123 and other pure ingredients, as determined by the XRD pattern (Figure 2F). This difference supported the idea that Dox was successfully entrapped in polymeric silica nanoparticles and that the amorphous quality of our final formulation promoted the drug-loading capacity [48].

### 3.2. Entrapment Efficiency and Loading Capacity of Dox in Dox/F127&P123-T_f_

Dox was effectively encapsulated into NPs, achieving respective EE and LC of 98.5 ± 4.4% and 22.6 ± 1.2% in Dox/F127&P123 and 95.7 ± 3.7% and 18.5 ± 2.5% in Dox/F127&P123-T_f_ (Figure 2G). The drug EE for Dox/F127&P123-T_f_ was slightly lower than that of Dox/F127&P123. This may be due to leaching of any residual drug during conjugation of T_f_ to the drug-loaded NPs. The LC of T_f_-conjugated NPs was also decreased compared to unconjugated NPs, further supporting the idea that drug was leached from conjugated NPs during the conjugation and incubation process [49,50].

### 3.3. In Vitro Drug Release Study

**Figure 3:**
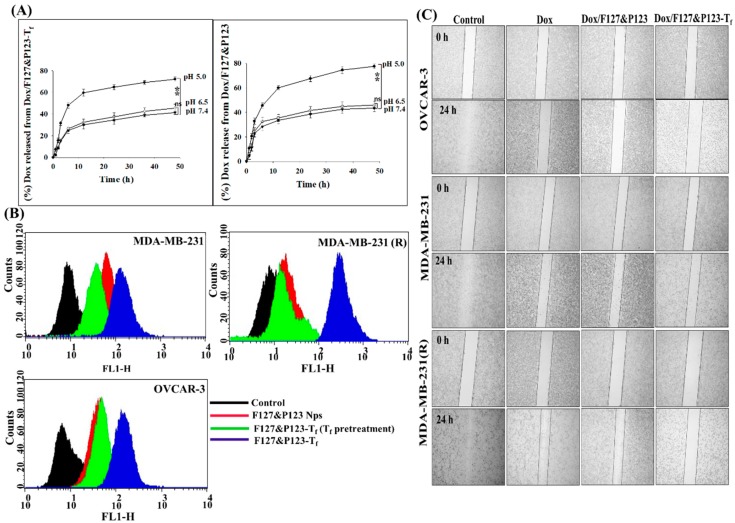
(**A**) In vitro release profiles of Dox from Dox/F127&P123 and Dox/F127&P123-T_f_ in ABS (pH 5.0) and PBS (pH 6.5 and 7.4) at 37 °C; data shown represent mean ± SD, (*n* = 6) (* *p* < 0.05, ** *p* < 0.01, *** *p* < 0.001); (**B**) cellular uptake of Dox/F127&P123-T_f_ with and without T_f_ pretreatment in OVCAR-3, MDA-MB-231, and MDA-MB-231(R); (**C**) effects of Dox, Dox/F127&P123, and Dox/F127&P123-T_f_ on wound healing and migration of OVCAR-3, MDA-MB-231, and MDA-MB-231(R); the images were observed under the optical microscope with 4× magnification at 0 h and 24 h; data shown represent mean ± SD (*n* = 3).

To estimate Dox release from Dox/F127&P123 and Dox/F127&P123-T_f_, in vitro drug release studies were carried out in both physiological media, phosphate-buffered saline (PBS, pH 6.5 and 7.4), and acidic media, acetate-buffered saline (ABS, pH 5.0). As shown in Figure 3A, the cumulative release of Dox from Dox/F127&P123 and Dox/F127&P123-T_f_ in acidic media was 89.5 ± 6.5% and 78.5 ± 7.5%, respectively, after 48 h. Both these values were higher than the rates of Dox release in PBS, which were 51.8 ± 2.8% (Dox/F127&P123) and 42.9 ± 3.5% (Dox/F127&P123-T_f_) at pH 7.4 and 58.5 ± 3.4% (Dox/F127&P123) and 44.4 ± 2.5% (Dox/F127&P123-T_f_) at pH 6.5. Modification of NPs with T_f_ may affect release rates to some degree, potentially due to the surface-shielding effects that allow for controlled release of drug and subsequent improvement of half-life [51].

### 3.4. In Vitro Cellular Uptake of Dox/F127&P123-T_f_

Fluorescence-activated cell sorting (FACS) analysis was performed on OVCAR-3, MDA-MB-231, and MDA-MB-231(R) cells to study the time- and concentration-dependent uptake patterns of Dox/F127&P123-T_f_ and compare these with the uptake patterns of Dox/F127&P123-T_f_ with and without T_f_ pretreatment. Enhancement in the uptake of Dox/F127&P123-T_f_ was evident with higher time and concentration of treatment in all cell lines (Figure S3). In particular, the increased uptake of Dox/F127&P123-T_f_ occurred even in the Dox-resistant cell line MDA-MB-231(R). In contrast, the uptake of T_f_-conjugated nanoparticles was significantly prohibited following T_f_ pretreatment because T_f_ receptors on cells had been blocked by free T_f_ molecules (Figure 3B). The uptake of Dox from Dox/F127&P123-T_f_ was successfully achieved with the aid of targeted PSNPs and the role receptor-mediated cellular uptake plays in targeted drug delivery has been corroborated by these results [52].

### 3.5. Cell Migration Efficacy of Dox/F127&P123-T_f_

Wound healing, scratch, and invasion assays were conducted to assess the progression of cancer cell development in both Dox-sensitive and -resistant cell lines, following treatment with free Dox and formulations with or without T_f_-targeting, and the results were compared with control. As shown in Figure 3C, all three cell lines treated with Dox/F127&P123-T_f_ invaded and migrated, but the antimigratory effect of Dox/F127&P123-T_f_ in MDA-MB-231(R) cells was significantly higher than that of free drug treatment and negative control. These results proved that our targeted drug-loaded nanoparticle can be used effectively in Dox-resistant cell lines [53].

### 3.6. In Vitro Cellular Cytotoxicity of Dox/F127&P123-T_f_

**Figure 4:**
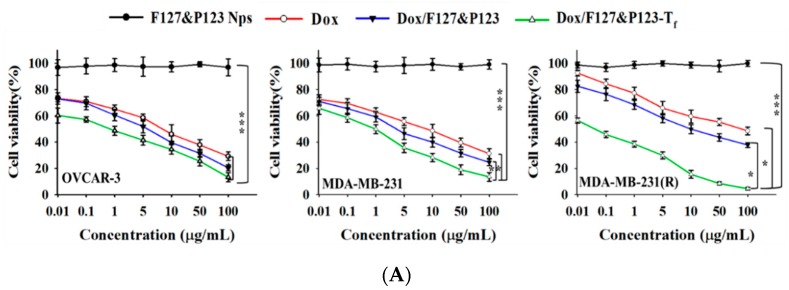

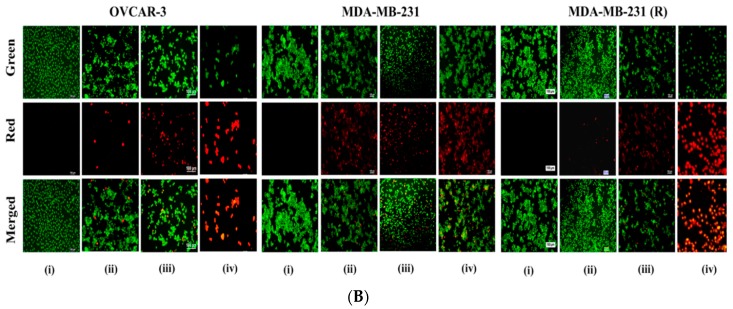
(**A**) In vitro cytotoxic effects of Dox, Dox/F127&P123, and Dox/F127&P123-T_f_ on OVCAR-3, MDA-MB-231, and MDA-MB-231(R) cells; data shown represent mean ± SD in three different experiments (*n* = 6) (* *p* < 0.05, ** *p* < 0.01, *** *p* < 0.001); (**B**) live/dead assay of OVCAR-3, MDA-MB-231, and MDA-MB-231(R) cells incubated with (ii) Dox, (iii) Dox/F127&P123, and (iv) Dox/F127&P123-T_f_ for 24 h, visualized with fluorescence microscopy; green and red fluorescence is defined as live and dead cells, (i) untreated group was used as control respectively; scale bars represent 100 µm.

The susceptibility to Dox/F127&P123-T_f_ of OVCAR-3, MDA-MB-231, and MDA-MB-231(R) cells was determined using the standard cell viability test, the MTT assay. The assay proved that the cytotoxic effect of Dox/F127&P123-T_f_ was higher than that of free Dox in not only the Dox-sensitive (OVCAR-3 and MDA-MB-231), but also the Dox-resistant MDA-MB-231(R) cell lines (Figure 4A). At the lowest dose (0.01 µg/mL) of Dox, Dox/F127&P123-T_f_ had greater cytotoxic effects in all three cell lines than free drug or unconjugated drug-loaded NPs. Among all three cell lines, MDA-MB-231(R) had the lowest cell viability following treatment with Dox/F127&P123-T_f_, suggesting that our T_f_-targeted Dox-loaded NPs inhibit cell proliferation of Dox-resistant breast cancer. This information was further proved by the IC_50_ values of the treated groups, calculated using the standard curve of each cell line (Figure S4, Table S1). The IC_50_ value of Dox/F127&P123-T_f_ was significantly lower than that of free Dox and drug-loaded nanoparticle without T_f_-conjugation. Moreover, in the Dox-resistant MDA-MB-231(R) cell line, the IC_50_ value of Dox/F127&P123-T_f_ was more significantly lower than other cell lines. The high IC_50_ value of free Dox in the MDA-MB-231(R) cell line confirmed that the cell line had successfully developed resistance. The mechanism underlying this weak potency is the removal of Dox from cells, following its uptake, via the ATP-dependent efflux transporters that are overexpressed in MDR cell lines [54].

### 3.7. Live and Dead Cell Assay of Dox/F127&P123-T_f_

The ability of nanoparticles to induce cell death and apoptosis was evaluated using a live/dead assay in all three cancer cell lines. Figure 4B shows viable (green) and dead (red) cells after 24 h of treatment with free Dox and formulations with/without T_f_-targeting. The intensity of red fluorescence for all three cell lines that treated with Dox/F127&P123-T_f_ was higher than that of non-targeted NPs and free drug, especially in the Dox-resistant MDA-MB-231(R) cell line. Therefore, the cytotoxic effect of targeted NPs must be more effective than non-targeted PSNPs not only in the Dox-sensitive cancer cell lines, but also in the resistant cell line, due to the specific receptor-mediated internalization of targeted NPs and increased cellular uptake of the drug [55].

### 3.8. Cell Cycle Arrest of Dox/F127&P123-T_f_

**Figure 5:**
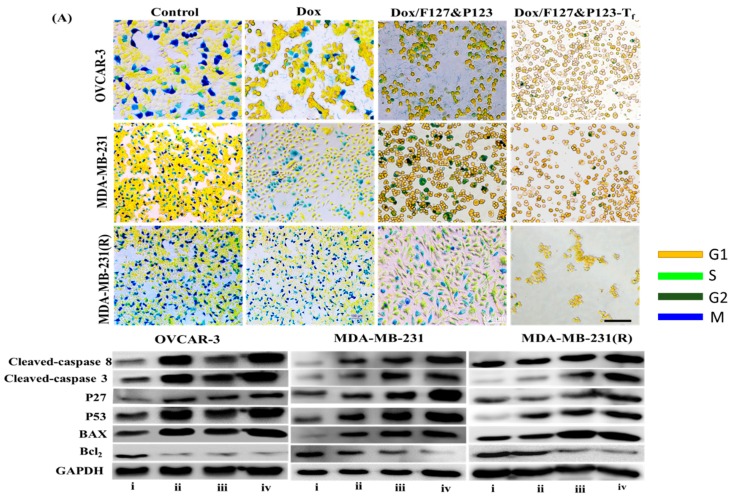
(**A**) Cell cycle analysis of OVCAR-3, MDA-MB-231, and MDA-MB-231(R) cells treated with Dox, Dox/F127&P123, and Dox/F127&P123-T_f_; scale bars represent 100 µm; (**B**) Western blot analysis of apoptotic and anti-apoptotic proteins induced in OVCAR-3, MDA-MB-231, and MDA-MB-231(R) cells treated with (ii) Dox, (iii) Dox/F127&P123 and (iv) Dox/F127&P123-T_f_ for 24 h.

As it is one of the fundamental methods for studying cellular apoptosis, cell cycle analysis was carried out in OVCAR-3, MDA-MB-231, and MDA-MB-231(R) cells; the results are presented in (Figure 5A and Figure S5). All three cell lines that were incubated with Dox/F127&P123-T_f_ showed lower populations in the G_2_/M phase, while the number of MDA-MB-231(R) cells treated with Dox in the G_2_/M phase was similar to that observed for the control group. Therefore, Dox/F127&P123-T_f_ inhibits mitosis (cell division) in Dox-sensitive and Dox-resistant cells by promoting the phase-specific mechanism of action of Dox, which affects cells in the S and G_2_ phases of the cell cycle due to higher cellular internalization in both the sensitive and resistant cancer cells [56].

### 3.9. Western Blot Analysis of Dox/F127&P123-T_f_

The levels of apoptotic and mitochondria-related proteins in OVCAR-3, MDA-MB-231, and MDA-MB-231(R) cells treated with Dox, Dox/F127&P123, and Dox/F127&P123-T_f_ were estimated via Western blot analysis. The comparative results of three cell lines are shown in (Figure 5B and Figure S6). The levels of caspase-3 and caspase-8 (fundamental protein markers in the apoptotic pathway), Bax (a mitochondria-related apoptotic marker [57]), and the cell cycle proteins p27 and p53 were significantly increased, while expression of the antiapoptotic protein Bcl-2 was significantly decreased, in Dox/F127&P123-T_f_-treated cell lines compared to cells treated with free Dox and non-targeted nanoparticle. These results definitively proved that Dox was successfully delivered not only to sensitive, but also to resistant cells by the targeted NPs. Dox/F127&P123-T_f_ increased the levels of p27 and p53, which disrupt of chromosome formation during mitosis, leading to cell cycle arrest at the G_2_/M phase and subsequent apoptosis. Moreover, Dox/F127&P123-T_f_ initiates downregulation of the antiapoptotic protein Bcl-2 through pores formed in the mitochondrial membranes of both sensitive and resistant cell lines [58].

### 3.10. In Vivo Imaging and Biodistribution Analysis

**Figure 6:**
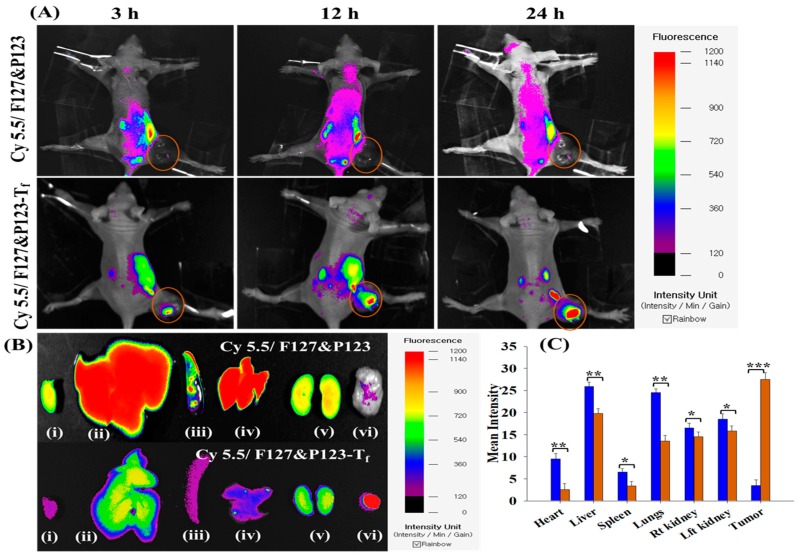
(**A**) In vivo biodistribution pattern of Cy5.5/F127&P123 and Cy5.5/F127&P123-T_f_ in MDA-MB-231(R) tumor-bearing mice; (**B**) ex vivo tissue distribution of Cy5.5/F127&P123 and Cy5.5/F127&P123-T_f_ in (i) heart, (ii) liver, (iii) spleen, (iv) lungs, (v) kidneys, and (vi) tumor excised from MDA-MB-231(R) tumor-bearing mice; (**C**) quantification of fluorescent signals from part B; data shown represent mean ± SD (*n* = 3).

The accumulation of Dox/F127&P123-T_f_ in xenograft mouse models bearing the T_f_-receptor expressing Dox-resistant cell line MDA-MB-231(R) was evaluated using an *in* vivo imaging apparatus and compared to that of cyanine 5.5 loaded non-targeted nanoparticles using an injection model of mice (Figure 6). The fluorescence intensity of cyanine 5.5 loaded targeted NPs was significantly higher than that of non-targeted NPs and there was minimal ex vivo distribution in the organs examined (heart, liver, spleen, kidney, and lungs) in the targeted NP treatment group. These results supported the successful preparation of a drug delivery system that more specifically targets tumor areas and avoids distribution in healthy organs.

## 4. Conclusions

The present study successfully developed a T_f_-targeted NP used as a Dox carrier into a drug-resistant cell line. Dox/F127&P123-T_f_ possesses several properties that will be useful for potential therapeutic applications, including small particle size, optimal surface charge to attach to cancer cells, and superior drug loading, while also allowing sustainable, controlled release of Dox. Moreover, Dox/F127&P123-T_f_ significantly enhanced cellular uptake and induced inhibition of cell proliferation in vitro, not only in Dox-sensitive cells, but also in the Dox-resistant cell line MDA-MB-231(R). In vitro data of Dox/F127&P123-T_f_ treatment in MDA-MB-231(R) suggest that drug resistance can be overcome by the accumulation of Dox in the nuclear region of the cancer cell via inhibition of P-gp mediated efflux. In addition, Dox/F127&P123-T_f_ successfully accumulated in xenograft mouse models bearing the Dox-resistant cell line MDA-MB-231(R), with minimum toxicity to healthy organs. Therefore, T_f_-targeted NPs can be used as safe and effective drug carriers for the treatment of both Dox-sensitive and -resistant tumors.

## Supplementary Materials

The following are available online at http://www.mdpi.com/1999-4923/11/2/63/s1, Figure S1. Characterization of implemented Dox-resistant MDA-MB-231(R) cells. (A) Cell viability of MDA-MB-231 and MDA-MB-231(R), (B) different proteins level (P-gp and P53) of MDA-MB-231 and MDA-MB-231(R), and (C) cellular uptake of Dox and Dox/F127&P123-T_f_ in MDA-MB-231 and MDA-MB-231(R). Figure S2. (A) FTIR spectrum and (B) ^1^H NMR analysis of carboxylated poloxamer F127. Figure S3. Comparison of time-dependent and concentration-dependent cellular uptake efficiency of Dox/F127&P123 in OVCAR-3, MDA-MB-231, and MDA-MB-231. Figure S4. Standard curve for calculation of IC 50 value of Dox and Dox/F127&P123-T_f_. Figure S5. Quantitative evaluation of comparison of cell cycle distribution of Dox, Dox/F127&P123 and Dox, Dox/F127&P123-T_f_ in OVCAR-3, MDA-MB231, and MDA-MB231(R). Figure S6. Quantitative evaluation of protein expression of (i) Cleaved-caspase 8, (ii) Cleaved-caspase 3, (iii) P27, (iv) P53, (v) BAX, and (vi) Bcl2 of P53 in OVCAR-3, MDA-MB231, and MDA-MB231 following treatment with Dox, Dox/F127&P123 and Dox, Dox/F127&P123-T_f_. Table S1. IC_50_ values of Dox and Dox/F127&P123 in three cell lines.
