# Supplementary material for: Transferrin-Conjugated Polymeric Nanoparticle for Receptor-Mediated Delivery of Doxorubicin in Doxorubicin-Resistant Breast Cancer Cells
Source: Pharmaceutics. 2019 Feb 1;11(2):63. doi: 10.3390/pharmaceutics11020063 (PMC6410246; doi:10.3390/pharmaceutics11020063)
Supplement: Supplementary file 1 [file pharmaceutics-11-00063-s001.pdf]

pharmaceutics

Supplementary Information: Transferrin-Conjugated Polymeric Nanoparticle for Receptor-Mediated Delivery of Doxorubicin in Doxorubicin-Resistant Breast Cancer Cells

Zar Chi Soe, Jun Bum Kwon, Raj Kumar Thapa, Wenquan Ou, Hanh Thuy Nguyen, Milan Gautam, Kyung Taek Oh, Han-Gon Choi, Sae Kwang Ku, Chul Soon Yong and Jong Oh Kim

Synthesis of carboxylated poloxamer F127

Carboxylated F127 was characterized by FTIR and proton nuclear magnetic resonance ^{1}H NMR analysis (Figure S2). According to its FTIR spectrum, the specific peaks such as C–O–C, O–H, C=O and methyl groups that are corresponding peaks of succinic anhydride and F127 occurred at (1159, 1630, 1735, and 2885) cm^{-1}. Moreover, carboxylated F127 was confirmed by peaks obtained from ^{1}H-NMR analysis.

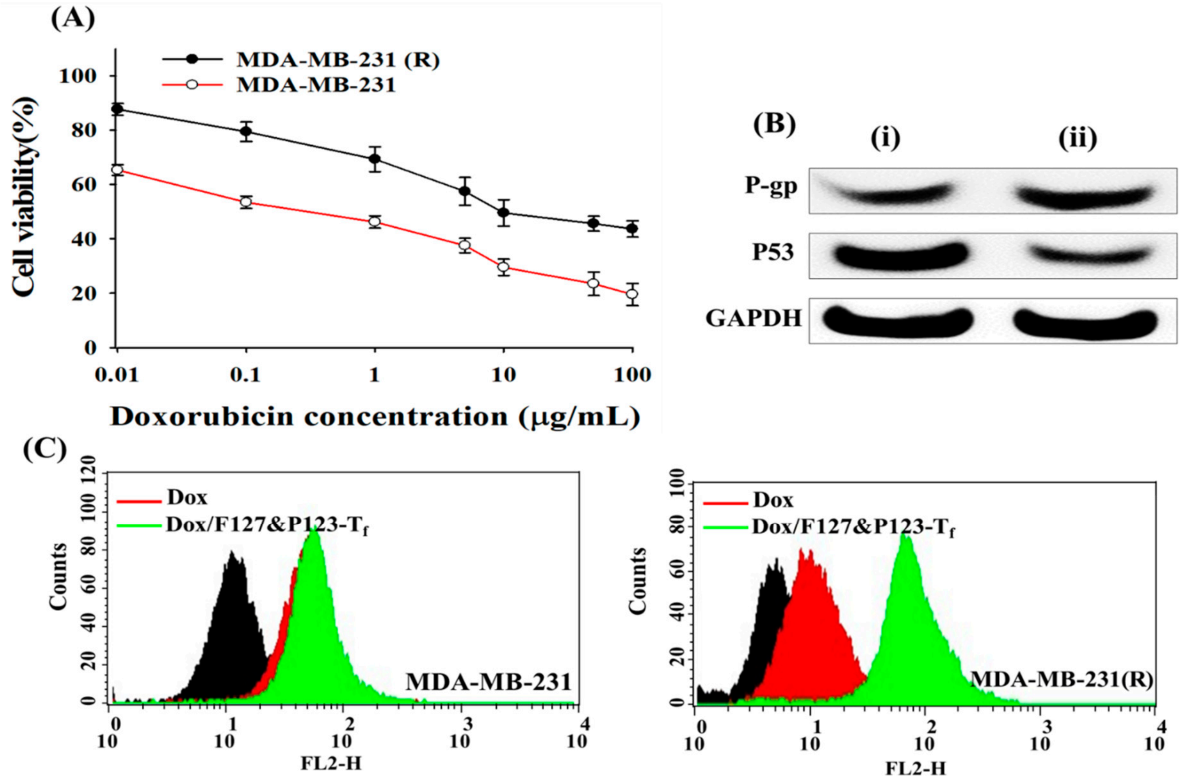

Figure S1. Characterization of implemented Dox-resistant MDA-MB-231(R) cells. (**A**) Cell viability of MDA-MB-231 and MDA-MB-231(R), (**B**) different proteins level (P-gp and P53) of MDA-MB-231 and MDA-MB-231(R), and (**C**) cellular uptake of Dox and Dox/F127&P123-T$_f$ in MDA-MB-231 and MDA-MB-231(R).

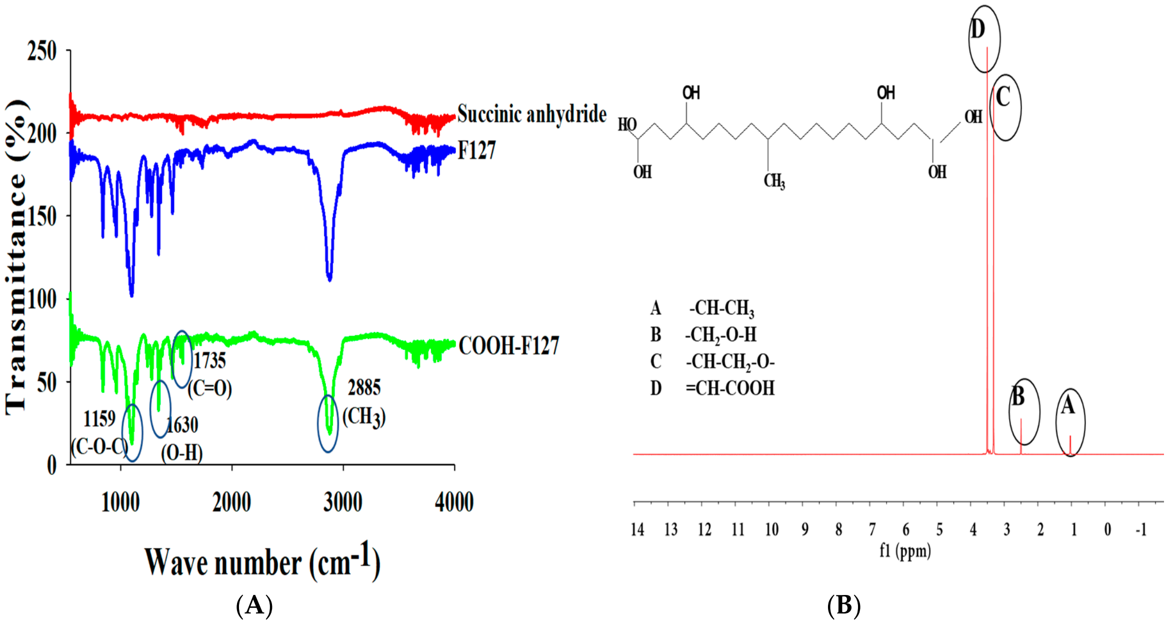

Figure S2. (**A**) FTIR spectrum and (**B**) ^{1}H NMR analysis of carboxylated poloxamer F127.

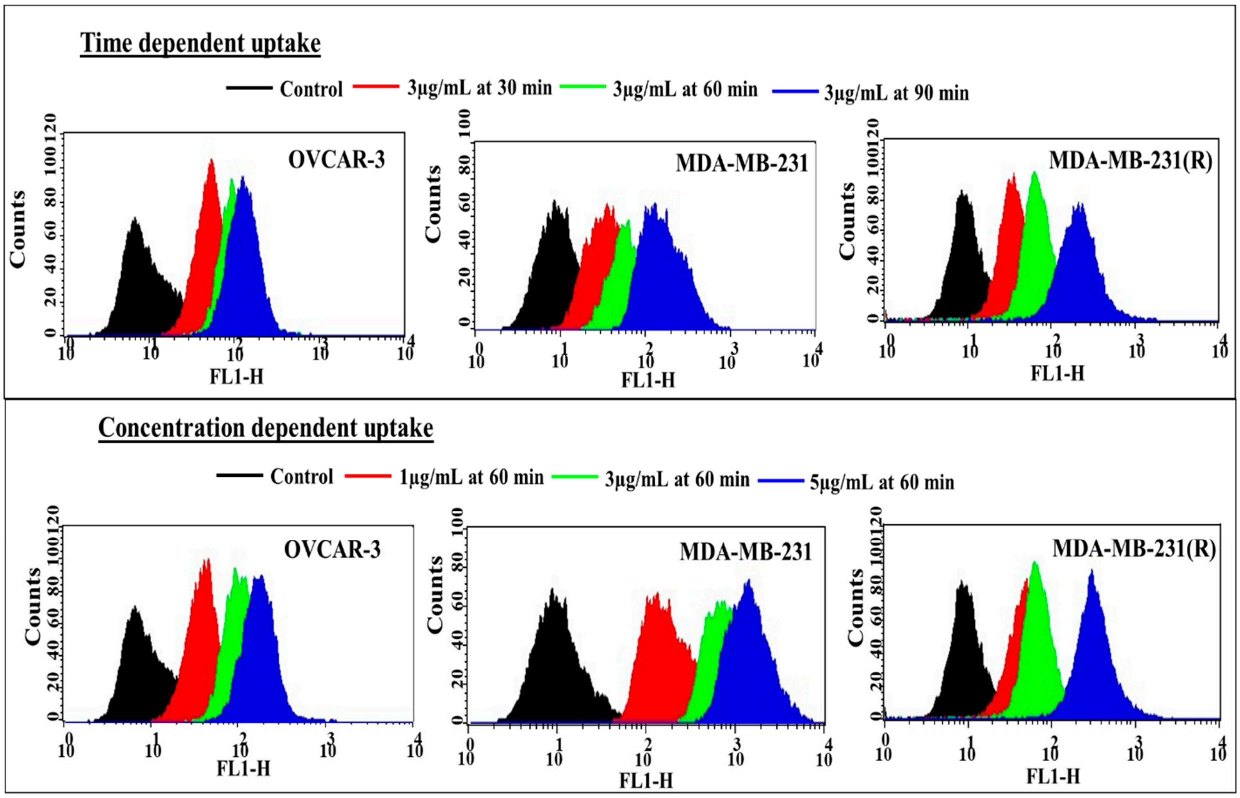

Figure S3. Comparison of time-dependent and concentration-dependent cellular uptake efficiency of Dox/F127&P123 in OVCAR-3, MDA-MB-231, and MDA-MB-231.

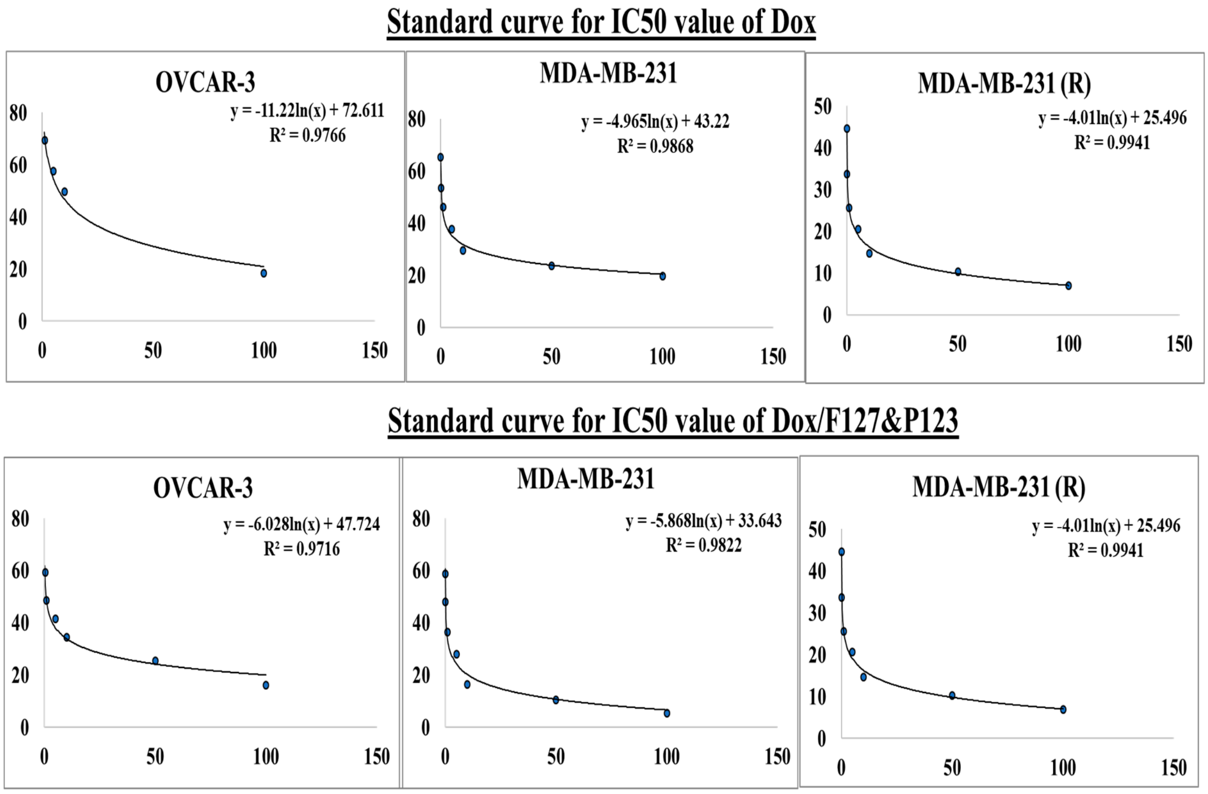

Figure S4. Standard curve for calculation of IC 50 value of Dox and Dox/F127&P123-Tf.

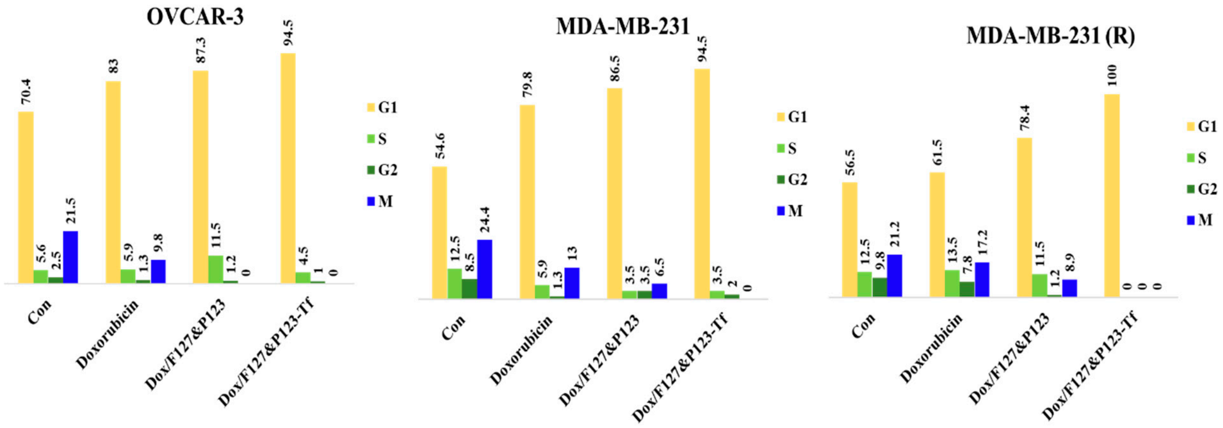

Figure S5. Quantitative evaluation of comparison of cell cycle distribution of Dox, Dox/F127&P123 and Dox, Dox/F127&P123-Tf in OVCAR-3, MDA-MB231, and MDA-MB231(R).

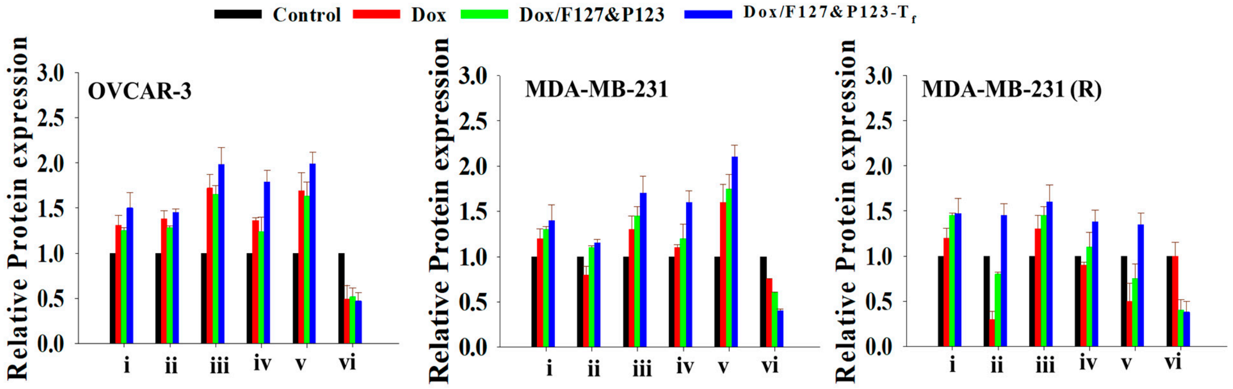

Figure S6. Quantitative evaluation of protein expression of (i) Cleaved-caspase 8, (ii) Cleaved-caspase 3, (iii) P27, (iv) P53, (v) BAX, and (vi) Bcl2 of P53 in OVCAR-3, MDA-MB231, and MDA-MB231 following treatment with Dox, Dox/F127&P123 and Dox, Dox/F127&P123-T_f.

Table S1. IC_{50} values of Dox and Dox/F127&P123 in three cell lines.

Cell lines	Dox (μg/mL)	Dox/F127&P123 (μg/mL)
OVCAR-3	8.5 ± 0.98	0.9 ± 0.05
MDA-MB-231	7.9 ± 1.24	1.6 ± 0.98
MDA-MB-231(R)	80.4 ± 1.85	0.1 ± 0.04
